# Effects of ambient air pollution on functional status in patients with chronic congestive heart failure: a repeated-measures study

**DOI:** 10.1186/1476-069X-6-26

**Published:** 2007-09-10

**Authors:** Gregory A Wellenius, Gloria Y Yeh, Brent A Coull, Helen H Suh, Russell S Phillips, Murray A Mittleman

**Affiliations:** 1 Cardiovascular Epidemiology Research Unit, Department of Medicine, Beth Israel Deaconess Medical Center, 330 Brookline Avenue, Boston, MA, 02215, USA; 2 Division of General Medicine and Primary Care, Department of Medicine, Beth Israel Deaconess Medical Center, 330 Brookline Avenue, Boston, MA, 02215, USA; 3 Department of Biostatistics, Harvard School of Public Health, 665 Huntington Avenue, Boston, MA 02115, USA; 4 Department of Environmental Health, Harvard School of Public Health, 665 Huntington Avenue, Boston, MA 02115, USA

## Abstract

**Background:**

Studies using administrative data report a positive association between ambient air pollution and the risk of hospitalization for congestive heart failure (HF). Circulating levels of B-type natriuretic peptide (BNP) are directly associated with cardiac hemodynamics and symptom severity in patients with HF and, therefore, serves as a marker of functional status. We tested the hypothesis that BNP levels would be positively associated with short-term changes in ambient pollution levels among 28 patients with chronic stable HF and impaired systolic function.

**Methods:**

BNP was measured in whole blood at 0, 6, and 12 weeks. We used linear mixed models to evaluate the association between fine particulate matter (PM_2.5_), carbon monoxide, sulfur dioxide, nitrogen dioxide, ozone, and black carbon and log(BNP). Lags of 0 to 3 days were considered in separate models. We calculated the intraclass correlation coefficient and within-subject coefficient of variation as measures of reproducibility.

**Results:**

We found no association between any pollutant and measures of BNP at any lag. For example, a 10 μg/m^3 ^increase in PM_2.5 _was associated with a 0.8% (95% CI: -16.4, 21.5; p = 0.94) increase in BNP on the same day. The within-subject coefficient of variation was 45% on the natural scale and 9% on the log scale.

**Conclusion:**

These results suggest that serial BNP measurements are unlikely to be useful in a longitudinal study of air pollution-related acute health effects. The magnitude of expected ambient air pollution health effects appears small in relation to the considerable within-person variability in BNP levels in this population.

## Background

Ambient air pollution is a recognized risk factor for cardiovascular morbidity and mortality [1]. Short-term elevations in ambient particulate matter have been specifically implicated in the triggering of acute cardiovascular events including myocardial infarction [2-4], ventricular arrhythmias [5-7], and ischemic stroke [8-10]. Several studies using administrative databases have shown a positive association between short-term increases in respirable or fine particles (particulate matter with aerodynamic diameter ≤10 μm (PM_10_) or ≤2.5 μm (PM_2.5_), respectively) and the risk of hospitalization for congestive heart failure (HF) [11-17]. There is emerging interest in evaluating the potential health effects of ambient pollution in patients with chronic stable HF by measuring within-patient changes in physiologic markers that represent intermediate endpoints [18], but we are not aware of any published studies.

Patients with HF who are hospitalized for acute symptom exacerbations characteristically have elevated cardiac filling pressures which promote fluid accumulation in the lungs and/or the peripheral tissues. B-type natriuretic peptide (BNP) is a neurohormone is produced principally by the ventricles of the heart in response to increasing wall stress. BNP has beneficial vasodilatory, natriuretic, and neurohormonal actions which act to reduce this wall stress [19]. Among patients undergoing treatment for acute decompensated HF, BNP levels are positively associated with symptom severity and key indices of cardiac hemodynamics [20,21], while administration of human recombinant BNP (Nesiritide) improves both hemodynamics and symptoms [22]. In patients with chronic stable HF, circulating levels are positively associated with all cause mortality [23].

If short-term increases in ambient pollution levels indeed increase the risk of hospitalization of HF patients for acute symptom exacerbation, it is plausible that associations might also be observed with more sensitive markers of functional status. Because of its favorable properties, BNP provides a measure of functional status in patients with HF and offers a potentially attractive opportunity for studying pollution-related health effects in this patient population. The goal of this study was to evaluate the association between short-term fluctuations in ambient air pollution concentrations and BNP levels in a panel of patients with chronic stable HF. To accomplish this, we carried out a retrospective analysis of a completed clinical trial in which 3 repeated BNP measurements were made in study participants over a 3 month period [24].

## Methods

This is a retrospective analysis of a completed clinical trial [24] that randomized 30 patients with HF and impaired systolic function to receive either 12 weeks of tai chi training in addition to their usual care, or to usual care alone. Patients were recruited from outpatient heart failure clinics at Beth Israel Deaconess Medical Center and Brigham and Women's Hospital in Boston, Massachusetts. Inclusion criteria included left ventricular ejection fraction ≤40% by echocardiography in the past year and maintenance on a stable medical regimen. The intervention consisted of 1-hour group tai chi classes held twice weekly for 12 weeks. Patients currently participating in conventional cardiac rehabilitation programs were excluded. Additional methodological details and results have been previously published [24].

BNP was measured at 0, 6 and 12 weeks from whole blood collected in ethylenediaminetetraacetic acid using a fluorescence immunoassay (Biosite Triage BNP Test; San Diego, California). Patients were followed between February 2002 and March 2003. BNP measurements were missing on 2 out of 84 (2.4%) visits.

We obtained daily measures of PM_2.5 _and black carbon from the Boston/Harvard Countway Library PM Center which is located <1 km from the study site. To reduce exposure misclassification, we excluded from analysis 2 patients who lived more than 40 km from this central monitor. Additionally, we obtained hourly measures of carbon monoxide (CO), nitrogen dioxide (NO_2_), sulfur dioxide (SO_2_), and ozone from the Massachusetts Department of Environmental Protection and calculated daily average values as previously described [6,7]. We obtained from the National Weather Service daily summaries of meteorological data measured at Logan International Airport.

We used linear mixed models with random subject-specific intercepts to evaluate the association between each pollutant and natural log-transformed BNP concentrations. We modeled each pollutant assuming a linear relationship and controlled for same day (lag 0) ambient temperature assuming a quadratic relationship, and mean ambient temperature over the past 3 days (lags 1–3), same day dew point, and mean dew point over the past 3 days assuming linear relationships with the outcome. Indicator variables for calendar month of blood draw, measurement occasion, treatment assignment, and measurement occasion by treatment assignment interactions were also included. PM_2.5 _levels at lags of 0 to 3 days were considered in separate models.

To assess the reproducibility of BNP and log(BNP) measurements over the 12 week period, we calculated the within-subject coefficient of variation (CV_WS_) and the intraclass correlation coefficient (ICC) among all subjects [25]. Variance components were estimated from a mixed model with random subject intercepts and a fixed effect for treatment assignment. As a sensitivity analysis, we repeated this analysis in the control group only. Analyses were carried out in SAS v9.1 (SAS Institute, Cary, NC). All p-values are based on two-sided tests at the α = 0.05 level. This analysis was granted an exemption from institutional review board review by the Beth Israel Deaconess Medical Center Committee on Clinical Investigations.

## Results

**Figure 1:**
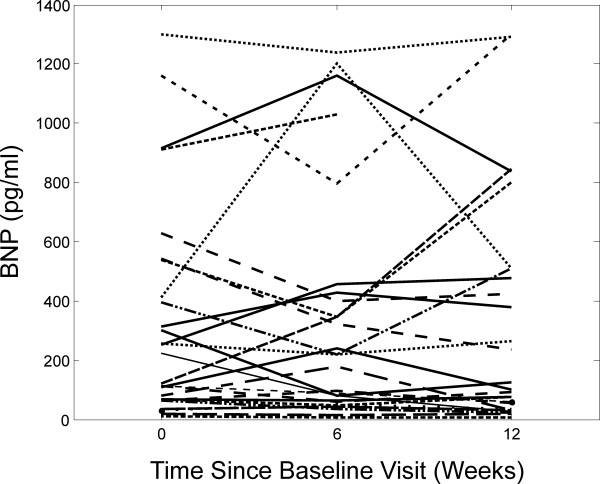
**BNP concentrations over time in 28 subjects with chronic stable HF**. Each line represents repeated measurements from a different subject.

Twenty-eight of the 30 patients enrolled in the randomized trial lived ≤40 km from the central monitoring site and were included in this analysis (Table 1). Subjects were predominantly male (64.3%) and white (53.6%). Age at baseline ranged from 33 to 88 years (64.3 ± 13.2; mean ± SD). Baseline left ventricular ejection fraction ranged from 10 to 35% (22.5 ± 7.1). BNP levels ranged from 5.7 to 1300 pg/ml, were log-normally distributed, and varied considerably within subjects (Fig. 1). Distributions over the entire study period of pollutant and meteorological variables are shown in Table 2. The subject-specific range (estimated as the difference between subject-specific maximum and minimum PM_2.5 _values at lag 0) varied from 0.7 to 50.9 μg/m^3 ^with a mean of 10.9 μg/m^3 ^and a median of 8.0 μg/m^3^, suggesting that PM_2.5 _levels varied substantially within subjects for the majority of participants.

**Table 1:** Patient characteristics at initial visit (mean ± SD or number (%)).

	Tai Chi Group (n = 14)	Control Group (n = 14)
Age (years)	66 ± 13	63 ± 14
Male Sex	9 (64)	9 (64)
Race		
Black	7 (50)	4 (29)
White	7 (50)	8 (57)
Asian	0	2 (14)
Ejection Fraction (%)	23.7 ± 6.4	21.2 ± 7.9
New York Heart Association Class		
I	3 (21)	1 (7)
II	6 (43)	8 (57)
III	3 (21)	5 (36)
IV	2 (14)	0
Heart Failure Etiology		
Idiopathic dilated	9 (64)	8 (57)
Ischemic	3 (21)	4 (29)
Hypertensive	0	1 (7)
Alcohol-related	1 (7)	1 (7)
Other	1 (7)	0
Weight (kg)	77.7 ± 19.7	92.2 ± 38.6
BNP (pg/ml)	344 ± 387	304 ± 346

**Table 2:** Distribution of pollutants and meteorological variables in the Boston Metropolitan area.^a^

Variable	N	Mean	SD	IQR	Correlation with PM_2.5_
PM_2.5 _(μg/m^3^)	345	10.9	8.4	8.1	--
Carbon Monoxide (ppm)	352	0.44	0.19	0.20	0.35
Sulfur Dioxide (ppb)	352	4.8	3.5	3.4	0.18
Nitrogen Dioxide (ppb)	352	20.7	5.9	7.8	0.31
Ozone (ppb)	352	25.1	12.9	15.2	0.35
Black Carbon (μg/m^3^)	352	0.73	0.45	0.49	0.68
Temperature (°C)	352	11.0	10.2	16.3	0.37
Dew Point (°C)	352	4.2	10.7	16.1	0.38
Barometric Pressure (mm Hg)	352	761.4	5.9	7.4	0.05

^a^Distribution is over the entire study period (February 28, 2002 – February 14, 2003). PM_2.5_: particulate matter with aerodynamic diameter ≤2.5 μm; IQR: interquartile range.

**Figure 2:**
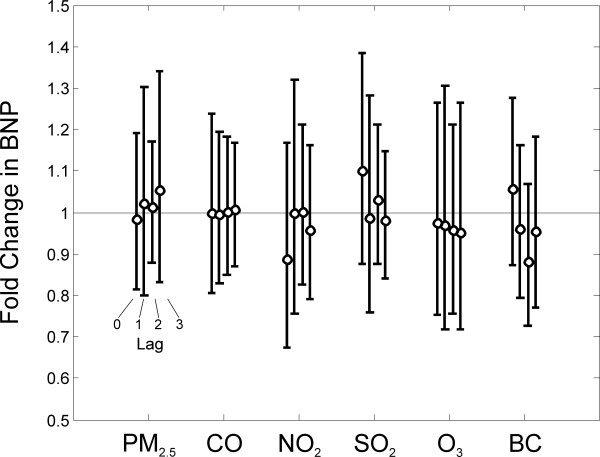
**Association between ambient air pollutants and BNP concentrations in 28 patients with chronic stable HF**. Results are from single-pollutant mixed models and represent the increase in BNP levels associated with an interquartile range increase in each pollutant, shown in Table 2.

PM_2.5 _levels were not significantly associated with a change in BNP levels at any of the lags examined. Specifically, an interquartile range increase (8.1 μg/m^3^) in PM_2.5 _at lags 0, 1, 2, and 3 days was associated with a -1.5% (95% CI: -18.7, 19.2), 2.1% (-20.0, 30.3), 1.3% (-12.3, 17.1), 5.6% (-16.8, 34.0) increase in BNP, respectively. No significant associations were observed between any other pollutant and BNP levels at any of the lags examined (Fig. 2).

We assessed the reproducibility of measures of BNP on the natural and log scales (Table 3). On the natural scale between-subject differences accounted for 85% of the total variance in measurements of BNP, as determined by the ICC. Although between-subject differences accounted for a large portion of the total variance, there was still substantial within-subject variability (CV_WS _= 45%). On the log scale, between-subject differences accounted for 89% of the total variance, and within-subject variability was more modest (CV_WS _= 9.0%). Limiting the analyses to those subjects in the control group improved the ICC and CV_WS _slightly.

**Table 3:** Reproducibility of BNP and log(BNP) over 12 weeks in patients with chronic stable HF.^a^

		Variance				
				
	Number of Subjects/Samples	Total	Between-Person	Within-Person	ICC	CV_WP_
BNP						
All Subjects	28/82	150,699	128,588	22,111	0.85	45.2%
Control Group Only	14/42	169,503	149,538	19,965	0.88	41.6%
Log(BNP)						
All Subjects	28/82	2.01	1.80	0.22	0.89	9.0%
Control Group Only	14/42	2.53	2.38	0.14	0.94	7.7%

^a ^HF: heart failure; ICC: intraclass correlation coefficient; CV_WP_: within-person coefficient of variation.

## Discussion

### Air Pollution and Heart Failure

Studies using administrative data have reported a positive association between short-term increases in ambient particles and the risk of hospitalization for HF [11-17]. While the magnitude of the effect is generally small (on the order of 1% increase in risk for a 10 μg/m^3 ^increase in PM), these estimates may be biased towards the null by extensive misclassification of both the exposure and the outcome. To address this problem, Symons et al. [26] evaluated this association using interview data obtained from 125 HF patients hospitalized after presenting to the emergency department and found no association between any pollutant and risk of symptom exacerbation or hospitalization.

However, none of the aforementioned studies have evaluated physiologic markers or intermediate endpoints repeatedly within subjects as has been extensively done in other patient populations including healthy elderly and those with a history of coronary artery disease, diabetes, or respiratory disease [reviewed by [27]]. Goldberg et al. recognized this gap in the literature and asked a panel of 31 HF patients aged ≥45 years to maintain a diary of their weight, blood oxygen saturation, pulse rate, and symptoms for 2 months. Preliminary results suggest a very small but statistically significant inverse association between ambient particles and blood oxygen saturation [18], but the final results have not yet been published. In the current study, we evaluated data from a cohort of patients with chronic stable HF and we found no evidence to suggest that short-term increases in ambient air pollutants are associated with changes in functional status as assessed by circulating levels of BNP.

These findings of very weak or no associations are disconcerting given the general consistency of the association between ambient pollution and risk of hospitalization for HF. One potential reason for the conflicting results is the heterogeneous clinical presentation and underlying pathophysiology of patients with HF. This heterogeneity is captured in the large administrative studies which identify cases according to discharge diagnoses, but not well captured in the more selective studies of Goldberg et al. [18], Symons et al. [26], or the current study where predefined inclusion and exclusion criteria are applied.

### Reproducibility of BNP Measurements

We observed that BNP measurements exhibited substantial variability. Although much of this variability was explained by differences between subjects, there remained substantial variability in BNP levels within individuals. There has been considerable interest in evaluating the use of single measurements of BNP and NT-pro-BNP (an inactive metabolite of pro-BNP) as a diagnostic aid for acute decompensated heart failure in patients presenting to the emergency department with acute dyspnea [21,28]. In contrast, the biologic variability – and hence the usefulness of serial BNP measurements in the same individual over time – has received relatively little attention [29-31]. Although small in size, prior studies have consistently shown that within-person hour-to-hour and day-to-day variability in the range of 15 – 30% should be expected in patients with stable HF. Only the study by Bruins et al. [30] followed subjects for more than 1 week. These investigators made repeated BNP measurements over a 6 week period in 43 patients with HF and found total within-person variability of 41%. This estimate compares favorably with the estimated CV_WS _of 45% from the current study.

Arguably, since our analyses of the effects of ambient pollution used log(BNP) as the outcome, CV_WS _of log(BNP) values is more relevant. We are not aware of any studies reporting the CV_WS _for BNP on the log scale. The CV_WS _provides an estimate of the within-subject variability relative to the mean response, and therefore is dependent on the measurement scale used. Thus, comparing the CV_WS _of BNP on the natural and log scales is not meaningful.

In the context of environmental exposures, these results imply that an impracticably large sample size would be required in a repeated-measures study to detect the typically modest effects of ambient air pollutants that one might expect to see at the level of an individual subject. In fact, based on the sample size formulas of Fitzmaurice et al. [32] and assuming that log(BNP) is modeled, in order to have 80% power to detect a 5.6% increase in BNP associated with an interquartile range increase in PM_2.5 _(the largest point estimate for PM_2.5 _observed in the current study), a longitudinal study with 5 bi-weekly measures per subject would require approximately 135 subjects. To put this effect size into perspective, one large clinical trial found that 4 months of treatment with the angiotensin receptor blocker valsartan reduced BNP levels by approximately 19% in patients with stable HF [33].

Alternative, more direct markers of cardiac function may perform better. For example, novel Doppler echocardiographic measures have been used in the context of pre-post studies to demonstrate that cigarette smoking acutely impairs ventricular relaxation during diastole in healthy volunteers [34,35]. By extension, serial echocardiography may provide better insight than BNP levels into the acute effects of ambient air pollution in patients with HF. However, the within-subject variability of these measures over periods of several weeks or months remains largely unknown.

### Strengths and Limitations

Potential limitations of this study include small sample size, only 3 repeated measures per subject, and an intervention that was shown to significantly lower BNP levels after 12 weeks. Other potential limitations are worth considering. First, we were not able to partition total within-person variability into analytic and biologic variability. However, for the BNP assay used in this study, the analytic coefficient of variation is 9–14%, which is small in comparison to the biologic variability. Second, we were not able to evaluate non-environmental sources of biologic variability such as changes in diet, health status, or medication usage. It is possible that accounting for these or other important time-varying factors would significantly reduce the apparent within-subject variability of BNP. Finally, this study specifically enrolled patients with HF and systolic dysfunction. Therefore, the results may not be generalizable to patients with HF and preserved systolic function. Strengths of this study include the proximity of the central site monitor to the study site, a small study area, and consistency of the measurements. Not withstanding the above limitations, we are not aware of any published studies that have assessed the effects of ambient air pollution on this novel biomarker.

## Conclusion

In this retrospective analysis of patients with HF enrolled in a completed clinical trial, we did not find an association between short-term fluctuations in ambient pollutant levels and functional status as assessed by circulating levels of BNP. Our results suggest that given the high within-person variability of this novel biomarker, BNP is unlikely to be a useful endpoint in the context of a repeated-measures study of the acute health effects of ambient air pollution.

## Competing interests

The author(s) declare that they have no competing interests.

## Authors' contributions

GAW contributed to study design and analysis, interpretation of the results, and manuscript preparation. GYY and RMP collected and provided access to original patient data and contributed to interpretation of the results. HHS contributed to exposure assessment and critical review of the manuscript BAC contributed to statistical analyses and critical review of the manuscript, MAM contributed to study design and analysis, interpretation of the results, and critical review of the manuscript.
